# Supplementary material for: The impact of the early COVID-19 pandemic on healthcare system resource use and costs in two provinces in Canada: An interrupted time series analysis
Source: PLoS One. 2023 Sep 8;18(9):e0290646. doi: 10.1371/journal.pone.0290646 (PMC10490868; doi:10.1371/journal.pone.0290646)
Supplement: S1 File — (DOCX) [file pone.0290646.s001.docx]

**The impact of the early COVID-19 pandemic on health care system resource use and costs: An interrupted time series analysis.**

**List of Supplementary material**

S1 Table. Administrative database descriptions in Ontario and British Columbia.

S1 Checklist. The RECORD statement – checklist of items, extended from the STROBE statement that should be reported in observational studies using routinely collected health data.

S2 Table. Definition of study outcomes and data sources used in each province.

S3 Table. Interrupted time series model and plot for use of physician services per 10,000 person-weeks in Ontario and British Columbia (BC). The parameter descriptions are in the notes below the table.

S4 Table. Interrupted time series model and plot for emergency department visits per 10,000 person-weeks in Ontario and British Columbia (BC). Please see the notes below S3 Table for parameter descriptions.

S5 Table. Interrupted time series model and plot for hospital admissions per 10,000 person-weeks in Ontario and British Columbia (BC). Please see the notes below S3 Table for parameter descriptions.

S6 Table. Interrupted time series model and plot for total healthcare costs per 10,000 person-weeks in Ontario and British Columbia (BC). Please see the notes below S3 Table for parameter descriptions.

S7 Table. Total and resource-specific absolute costs (2020 Canadian dollars) per quarter for all individuals in Ontario, 2018 and 2019. For readability, all costs are rounded to the nearest thousand dollars.

S8 Table. Total and resource-specific absolute costs (2020 Canadian dollars) per quarter for all individuals in British Columbia (BC), 2018 and 2019. For readability, all costs are rounded to the nearest thousand dollars.

S9 Table. Total absolute costs (in 2020 Canadian dollars) in 2020 for the full population and for individuals who tested positive for SARS-Cov-2 in Ontario and British Columbia (BC). For readability, all costs are rounded to the nearest thousand dollars.

S1 Figure. Number of cancer clinic visits in Ontario per 10,000 person-weeks in each week from January 1, 2018 to December 27, 2020. The vertical line indicates the week of March 16 to 22, 2020.

S2 Figure. Number of a) home care services in Ontario and British Columbia, and b) dialysis clinic visits in Ontario per 10,000 person-weeks in each week from January 1, 2018 to December 27, 2020. The vertical line indicates the week of March 16 to 22, 2020.

**S1 Table. Administrative database descriptions in Ontario and British Columbia**

| Resource | Ontario | British Columbia (BC) | Description |
| --- | --- | --- | --- |
| Cohort characteristics | Registered Persons Database [1] | Medical Services Plan (MSP) Consolidation file [2] | Demographic information on residents enrolled in the provincial health insurance plan (OHIP in Ontario; MSP in BC) |
| COVID testing | C19INTGR [3] | COVID-19 Test Lab data [4] | Laboratory test requests and results for COVID-19 (SARS-CoV-2) using real-time polymerase chain reaction testing. |
| Hospital separations | Canadian Institute for Health Information – Discharge Abstract Database (CIHI-DAD) [5] | Canadian Institute for Health Information – Discharge Abstract Database (CIHI-DAD) [6] | Information on all inpatient hospital discharges, including clinical and demographic information. Each record has 25 fields for acute and chronic diagnoses, coded using ICD-10-CA. Dates of admission to intensive care units are also recorded in the hospital discharge records. |
| Same-day surgery visits | CIHI- National Ambulatory Care Reporting System (CIHI- NACRS same-day surgery [7] | CIHI- Discharge Abstract Database (CIHI-DAD) [6] | Information on visits to same-day surgery clinics, with diagnoses coded using ICD-10-CA and procedures coded using the Canadian Classifications of Health Interventions (CCI). |
| Ambulatory care | CIHI-NACRS [7] | CIHI –NACRS [8] | Information on all hospital and community-based ambulatory care clinics, including emergency department visits (Ontario and BC). Visits to dialysis clinics and cancer clinics (at cancer centre and hospitals), and other hospital clinics are included in Ontario. |
| Outpatient prescription drugs | Ontario Drug Benefit Claims (ODB) [9] | PharmaNet [10] | Eligible individuals for the ODB include those aged 65 and older, residents of LTC homes and homes for special care, and recipients of professional home care services, social assistance services, and the Trillium Drug Program. A flag in each prescription record identifies prescriptions provided to LTC residents.  PharmaNet data capture all outpatient prescription drug claims dispensed in community pharmacies and hospital outpatient pharmacies in BC, and their cost. |
| Physician services | Ontario Health Insurance Plan (OHIP) Claims Database [11] | Medical Services Plan (MSP) payment information file [12] | The OHIP database contains claims submitted by physicians with a fee code that describes the service provided. Office visits and services provided in ambulatory clinics, LTC facilities, and acute care and chronic care hospitals are included, as well as house calls, telephone visits and virtual visits. OHIP-covered non-physician health care practitioners can submit claims under specific circumstances.  Records of medically necessary laboratory tests performed at community laboratories are included in the OHIP database.  The MSP file contains data on all medically necessary services provided to individuals under BC’s universal health insurance program. |
| Home care services | Home Care Database [13] | See row below | Records of services provided in the home, in-person or by phone. Records include diagnosis and service provided. |
| Long-term care (LTC) services | CIHI - Continuing Care Reporting System (CCRS) [14] | Home and Community care -Minimum Reporting Requirements database (HCC-MRR) [15] | The CCRS contains demographic, clinical, functional and resource utilization information on people receiving continuing care services in hospitals or LTC facilities.  The HCC-MRR contains demographic and resource utilization data on publicly-funded clients of LTC facilities and home care. |
| New Drug Funding Program (NDFP)  chemotherapy | New Drug Funding Program database [16] |  | The New Drug Funding Program reimburses cancer centres for expensive intravenous cancer chemotherapy (Ontario only). |

**S1 Checklist:** The RECORD statement – checklist of items, extended from the STROBE statement that should be reported in observational studies using routinely collected health data [17]

|  | **Item No.** | **STROBE items** | **Location in manuscript where items are reported** | **RECORD items** | **Location in manuscript where items are reported** |
| --- | --- | --- | --- | --- | --- |
| **Title and abstract** | | | | | |
|  | 1 | (a) Indicate the study’s design with a commonly used term in the title or the abstract  (b) Provide in the abstract an informative and balanced summary of what was done and what was found | (a) Abstract    (b) YES | RECORD 1.1: The type of data used should be specified in the title or abstract. When possible, the name of the databases used should be included.  RECORD 1.2: If applicable, the geographic region and timeframe within which the study took place should be reported in the title or abstract.  RECORD 1.3: If linkage between databases was conducted for the study, this should be clearly stated in the title or abstract. | 1.1 Abstract  1.2 Title; Abstract  1.3 Abstract |
| **Introduction** | | | | | |
| Background rationale | 2 | Explain the scientific background and rationale for the investigation being reported | Introduction |  |  |
| Objectives | 3 | State specific objectives, including any prespecified hypotheses | Introduction |  |  |
| **Methods** | | | | | |
| Study Design | 4 | Present key elements of study design early in the paper | Data sources and settings, and Study design and inclusion criteria subsections |  |  |
| Setting | 5 | Describe the setting, locations, and relevant dates, including periods of recruitment, exposure, follow-up, and data collection | Data sources and settings, and Study design and inclusion criteria subsections |  |  |
| Participants | 6 | *(a) Cohort study* - Give the eligibility criteria, and the sources and methods of selection of participants. Describe methods of follow-up  *Case-control study* - Give the eligibility criteria, and the sources and methods of case ascertainment and control selection. Give the rationale for the choice of cases and controls  *Cross-sectional study* - Give the eligibility criteria, and the sources and methods of selection of participants  *(b) Cohort study* - For matched studies, give matching criteria and number of exposed and unexposed  *Case-control study* - For matched studies, give matching criteria and the number of controls per case | Study design and inclusion criteria subsection | RECORD 6.1: The methods of study population selection (such as codes or algorithms used to identify subjects) should be listed in detail. If this is not possible, an explanation should be provided.  RECORD 6.2: Any validation studies of the codes or algorithms used to select the population should be referenced. If validation was conducted for this study and not published elsewhere, detailed methods and results should be provided.  RECORD 6.3: If the study involved linkage of databases, consider use of a flow diagram or other graphical display to demonstrate the data linkage process, including the number of individuals with linked data at each stage. | 6.1 Study design and inclusion criteria subsection  6.2 Study design and inclusion criteria subsection  6.3 not applicable |
| Variables | 7 | Clearly define all outcomes, exposures, predictors, potential confounders, and effect modifiers. Give diagnostic criteria, if applicable. | Study outcomes and statistical analysis subsection | RECORD 7.1: A complete list of codes and algorithms used to classify exposures, outcomes, confounders, and effect modifiers should be provided. If these cannot be reported, an explanation should be provided. | 7.1 S1 Table and S2 Table |
| Data sources/ measurement | 8 | For each variable of interest, give sources of data and details of methods of assessment (measurement).  Describe comparability of assessment methods if there is more than one group | S1 Table and S2 Table |  |  |
| Bias | 9 | Describe any efforts to address potential sources of bias | Not applicable |  |  |
| Study size | 10 | Explain how the study size was arrived at | Study design and inclusion criteria subsection |  |  |
| Quantitative variables | 11 | Explain how quantitative variables were handled in the analyses. If applicable, describe which groupings were chosen, and why | Study outcomes and statistical analysis subsection |  |  |
| Statistical methods | 12 | (a) Describe all statistical methods, including those used to control for confounding  (b) Describe any methods used to examine subgroups and interactions  (c) Explain how missing data were addressed  (d) *Cohort study* - If applicable, explain how loss to follow-up was addressed  *Case-control study* - If applicable, explain how matching of cases and controls was addressed  *Cross-sectional study* - If applicable, describe analytical methods taking account of sampling strategy  (e) Describe any sensitivity analyses | (a) Study outcomes and statistical analysis subsection  (b) Study outcomes and statistical analysis subsection  (c) not applicable  (d) not applicable  (e) not applicable |  |  |
| Data access and cleaning methods |  | .. |  | RECORD 12.1: Authors should describe the extent to which the investigators had access to the database population used to create the study population.  RECORD 12.2: Authors should provide information on the data cleaning methods used in the study. | 12.1 Data sources and settings subsection  12.2 not applicable |
| Linkage |  | .. |  | RECORD 12.3: State whether the study included person-level, institutional-level, or other data linkage across two or more databases. The methods of linkage and methods of linkage quality evaluation should be provided. | 12.3 Data sources and settings subsection |
| **Results** | | | | | |
| Participants | 13 | (a) Report the numbers of individuals at each stage of the study (*e.g.*, numbers potentially eligible, examined for eligibility, confirmed eligible, included in the study, completing follow-up, and analysed)  (b) Give reasons for non-participation at each stage.  (c) Consider use of a flow diagram | (a) not applicable  (b) not applicable  (c) not applicable | RECORD 13.1: Describe in detail the selection of the persons included in the study (*i.e.,* study population selection) including filtering based on data quality, data availability and linkage. The selection of included persons can be described in the text and/or by means of the study flow diagram. | 13.1 Description of study populations subsection |
| Descriptive data | 14 | (a) Give characteristics of study participants (*e.g.*, demographic, clinical, social) and information on exposures and potential confounders  (b) Indicate the number of participants with missing data for each variable of interest  (c) *Cohort study* - summarise follow-up time (*e.g.*, average and total amount) | (a) Description of study populations subsection and Table 1  (b) not applicable  (c) not applicable |  |  |
| Outcome data | 15 | *Cohort study* - Report numbers of outcome events or summary measures over time  *Case-control study* - Report numbers in each exposure category, or summary measures of exposure  *Cross-sectional study* - Report numbers of outcome events or summary measures | Results section after Description of study populations subsection, including Tables 2 and 3; Figures 1 to 3; S3 Table to S9 Table; S1 Figure and S2 Figure |  |  |
| Main results | 16 | (a) Give unadjusted estimates and, if applicable, confounder-adjusted estimates and their precision (e.g., 95% confidence interval). Make clear which confounders were adjusted for and why they were included  (b) Report category boundaries when continuous variables were categorized  (c) If relevant, consider translating estimates of relative risk into absolute risk for a meaningful time period | (a) Resource use by category subsection, Healthcare costs subsection, and Tables 2 and 3  (b) Table 1  (c) not applicable |  |  |
| Other analyses | 17 | Report other analyses done—e.g., analyses of subgroups and interactions, and sensitivity analyses | Direct healthcare costs for COVID-19 subsection, and S9 Table |  |  |
| **Discussion** | | | | | |
| Key results | 18 | Summarise key results with reference to study objectives | Discussion, first paragraph and last paragraph before Conslusions |  |  |
| Limitations | 19 | Discuss limitations of the study, taking into account sources of potential bias or imprecision. Discuss both direction and magnitude of any potential bias | Discussion, Strengths and limitations subsection | RECORD 19.1: Discuss the implications of using data that were not created or collected to answer the specific research question(s). Include discussion of misclassification bias, unmeasured confounding, missing data, and changing eligibility over time, as they pertain to the study being reported. | 19.1 Discussion, Strengths and limitations subsection |
| Interpretation | 20 | Give a cautious overall interpretation of results considering objectives, limitations, multiplicity of analyses, results from similar studies, and other relevant evidence | Conclusions |  |  |
| Generalisability | 21 | Discuss the generalisability (external validity) of the study results | Discussion, second to fourth paragraphs |  |  |
| **Other Information** | | | | | |
| Funding | 22 | Give the source of funding and the role of the funders for the present study and, if applicable, for the original study on which the present article is based | Acknowledgements,  and Funding information |  |  |
| Accessibility of protocol, raw data, and programming code |  | N/A |  | RECORD 22.1: Authors should provide information on how to access any supplemental information such as the study protocol, raw data, or programming code. | 22.1 Data availability statements |

*Checklist is protected under Creative Commons Attribution ([CC BY](http://creativecommons.org/licenses/by/4.0/)) license.

**S2 Table. Definition of study outcomes and data sources used in each province**

| Study Outcomes | Data sources | Description |
| --- | --- | --- |
| Overall health care resources costs | Linkages of all data sources using unique encoded identifiers in each province separately. | The weekly sum of costs for hospitalization, emergency department visits, outpatient clinics (Ontario), physician services, laboratory tests, home and community care and outpatient prescription drugs over 10,000 person-weeks. |
| Physicians’ services rate | OHIP in Ontario  MSP billing in BC | The weekly number of physician services over 10,000 person-weeks. |
| Hospitalization admissions rate | CIHI-DAD in Ontario and BC | The weekly number of hospital admissions (acute care only) over 10,000 person-weeks. |
| Emergency department visits rate | NACRS in Ontario and BC | The weekly number of emergency department visits over 10,000 person-weeks. |
| Home and community care visits rate | Home Care Database in Ontario;  Home and Community care -Minimum Reporting Requirements database (HCC-MRR) in BC | The weekly number of services received in home and community care over 10,000 person-weeks. |
| Cancer clinic visits rate | NACRS (Ontario only) | The weekly number of visits to cancer clinics per 10,000 person weeks. |
| Dialysis clinics visits rate | NACRS (Ontario only) | The weekly number of visits to dialysis clinics per 10,000 person-weeks. |

Abbreviations- BC: British Columbia; CIHI-DAD: Canadian Institute for Health Information –Discharge Abstract Database; HCC-MRR: Home and Community care -Minimum Reporting Requirements database; MSP: Medical Services Plan; NACRS: National Ambulatory Care Reporting System; OHIP: Ontario Health Insurance Plan

**S3 Table.** Interrupted time series (ITS) model and plot for use of physician services per 10,000 person-weeks in Ontario and British Columbia (BC). Please see notes below table for parameter descriptions.

Ontario as reference

| Parameter | Estimate | Standard error | p-value |
| --- | --- | --- | --- |
| intercept | 3342.25 | 35.69 | <0.001 |
| post | -1437.42 | 91.06 | <0.001 |
| province BC | -1138.33 | 42.11 | <0.001 |
| time- pre-pandemic | 0.11 | 0.39 | 0.787 |
| wild | 1359.71 | 93.24 | <0.001 |
| lag 1 | 34.09 | 16.54 | 0.041 |
| poly (time post, 2) 1 | 8053.22 | 568.46 | <0.001 |
| poly (time post, 2) 2 | -1617.84 | 310.30 | <0.001 |
| holiday- last week of December | -1463.92 | 112.62 | <0.001 |
| holiday- first week of January | -1885.75 | 119.59 | <0.001 |
| holiday- other statutory | -302.61 | 35.31 | <0.001 |
| province BC: post | 919.42 | 98.58 | <0.001 |
| province BC: time pre-pandemic | -0.56 | 0.55 | 0.310 |
| province BC: wild | 784.60 | 119.04 | <0.001 |
| province BC: poly (time post, 2) 1 | -4227.52 | 645.03 | <0.001 |
| province BC: poly (time post, 2) 2 | 669.46 | 402.63 | 0.098 |
| province BC: holiday- last week of December | 432.09 | 118.49 | <0.001 |
| province BC: holiday- first week of January | 1501.50 | 117.34 | <0.001 |
| province BC: holiday- other statutory | 81.58 | 43.43 | 0.062 |

BC as reference

| Parameter | Estimate | Standard error | p-value |
| --- | --- | --- | --- |
| intercept | 2203.92 | 34.75 | <0.001 |
| post | -517.99 | 87.24 | <0.001 |
| province Ontario | 1138.33 | 42.11 | <0.001 |
| time- pre-pandemic | -0.45 | 0.40 | 0.251 |
| wild | -575.99 | 91.86 | <0.001 |
| lag 1 | 34.09 | 16.54 | 0.041 |
| poly (time post, 2) 1 | 3825.69 | 582.02 | <0.001 |
| poly (time post, 2) 2 | -948.38 | 313.47 | 0.003 |
| holiday- last week of December | -1031.83 | 112.66 | <0.001 |
| holiday- first week of January | -384.25 | 130.99 | 0.004 |
| holiday- other statutory | -221.03 | 35.30 | <0.001 |
| province Ontario: post | 919.42 | 98.58 | <0.001 |
| province Ontario: time pre | 0.56 | 0.55 | 0.310 |
| province Ontario: wild | -784.60 | 119.04 | <0.001 |
| province Ontario: poly (time post, 2) 1 | 4227.52 | 645.03 | <0.001 |
| province Ontario: poly (time post, 2) 2 | -669.46 | 402.63 | 0.098 |
| province Ontario: holiday- last week of December | -432.09 | 118.49 | <0.001 |
| province Ontario: holiday- first week of January | -1501.50 | 117.34 | <0.001 |
| province Ontario: holiday- other statutory | -81.58 | 43.43 | 0.062 |

**Parameter descriptions**

**Intercept**- Expected value for the final pre-COVID-19 week

**Post** - Post-COVID-19 week 1 versus final pre-COVID-19 week

**Province BC/Province Ontario** - BC versus Ontario/Ontario versus BC

**Time- pre-pandemic** - change over time per week in the pre-pandemic period, from January 1, 2018 to March 15, 2020

**Wild** - The first 3 weeks of the pandemic period (March 16, 2020 to April 5, 2020)

**Lag 1** - the value for the previous week (to reduce autocorrelation)

**poly (time post, 2) 1** - first order (linear term) in the post-pandemic period

**poly (time post, 2) 2** - second order (quadratic term) in the post-pandemic period

**holiday- last week of December** - change associated with the last week of December holidays

**holiday- first week of January** - change associated with the first week of January holidays

**holiday- other statutory** - change associated with other statutory holidays

**Province BC/Province** Ontario - Time- pre-pandemic - difference in the change over time per week in the pre-pandemic period, from January 1, 2018 to March 15, 2020, in BC versus Ontario/Ontario versus BC

**Province BC/Province Ontario: Wild** - difference in Wild in BC versus Ontario/Ontario versus BC

**Province BC/Province Ontario: Lag 1** - difference in Lag 1 in BC versus Ontario/Ontario versus BC

**Province BC/Province Ontario: poly (time post, 2) 1** - difference in poly (time post, 2) 1 in BC versus Ontario/Ontario versus BC

**Province BC/Province Ontario: poly (time post, 2) 2** - difference in poly (time post, 2) 2 in BC versus Ontario/Ontario versus BC

**Province BC/Province Ontario: holiday- last week of December** - difference in the change associated with the last week of December holidays in BC versus Ontario/Ontario versus BC

**Province BC/Province Ontario: holiday- first week of January** - difference in the change associated with the first week of January holidays in BC versus Ontario/Ontario versus BC

**Province BC/Province Ontario: holiday- other statutory** - difference in the change associated with other statutory holidays in BC versus Ontario/Ontario versus BC

Each of the above terms was allowed to interact with an indicator variable for province, which afforded separate estimates for each province while fitting only one generalized linear mixed model per outcome.

Interrupted time series (ITS) plot for physician services in Ontario and British Columbia (BC)


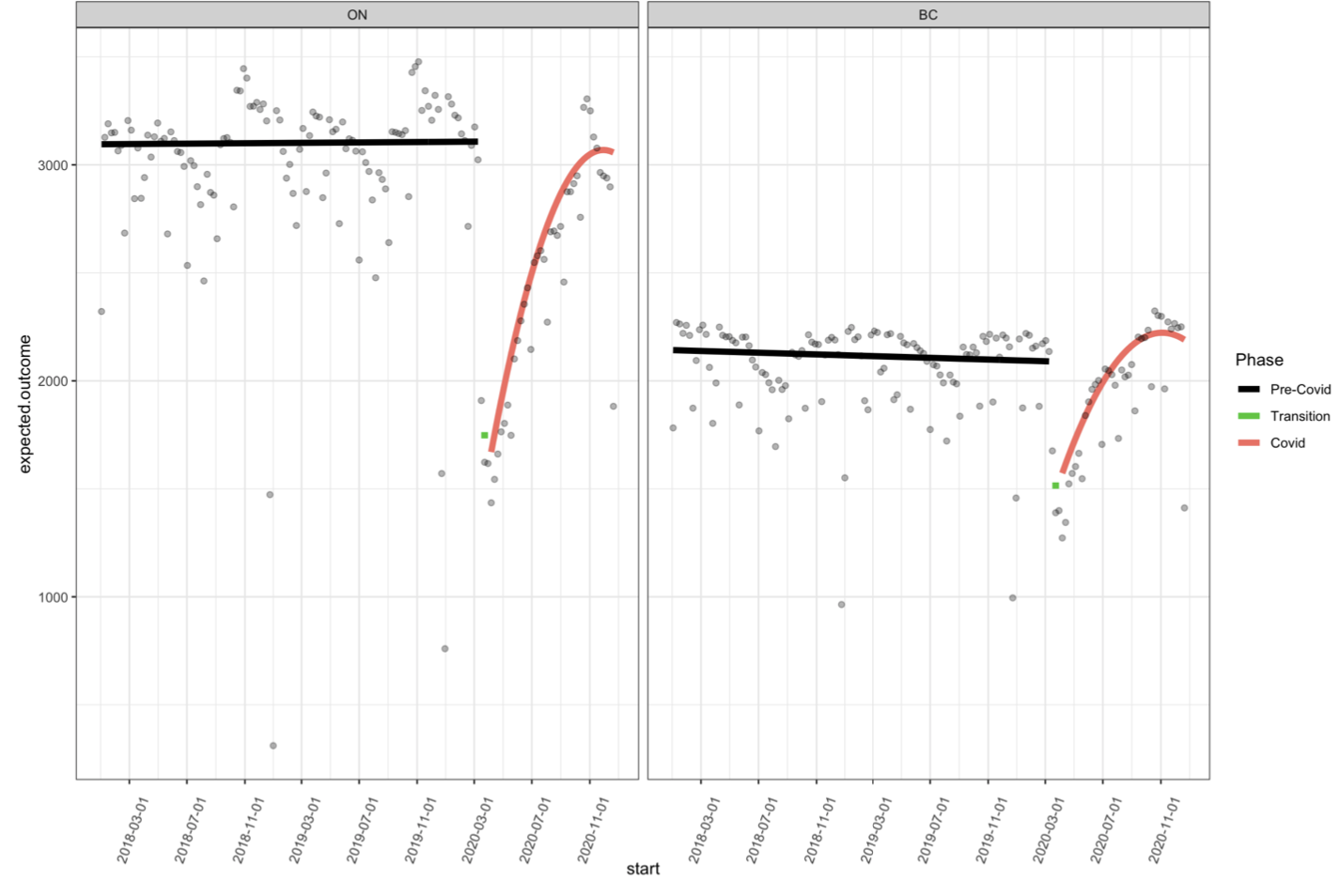


Notes: The figure shows weekly data points and linear mixed effects regression (LMER)-based expected values, conditional on the estimated ISO week level random intercepts.

The transition point represents the “wild” coefficient.

S4 Table. Interrupted time series model and plot for emergency department visits per 10,000 person-weeks in Ontario and British Columbia (BC). Please see the notes below S1 Table for parameter descriptions.

Ontario as reference

| Parameter | Estimate | Standard error | p-value |
| --- | --- | --- | --- |
| intercept | 81.46 | 0.81 | <0.001 |
| post | -34.01 | 2.27 | <0.001 |
| province BC | -19.98 | 1.09 | <0.001 |
| time- pre-pandemic | -0.05 | 0.01 | <0.001 |
| wild | -31.06 | 2.28 | <0.001 |
| lag 1 | 1.32 | 0.41 | 0.001 |
| poly (time post, 2) 1 | 140.54 | 13.36 | <0.001 |
| poly (time post, 2) 2 | -98.35 | 8.26 | <0.001 |
| holiday- last week of December | 8.02 | 2.17 | <0.001 |
| holiday- first week of January | -41.29 | 2.12 | <0.001 |
| holiday- other statutory | 0.33 | 35.31 | 0.674 |
| province BC: post | 16.88 | 2.55 | <0.001 |
| province BC: time pre | 0.04 | 0.01 | 0.005 |
| province BC: wild | 9.77 | 3.09 | 0.002 |
| province BC: poly (time post, 2) 1 | -75.00 | 16.66 | <0.001 |
| province BC: poly (time post, 2) 2 | 49.21 | 14.41 | <0.001 |
| province BC: holiday- last week of December | -3.28 | 3.06 | 0.285 |
| province BC: holiday- first week of January | 45.13 | 2.96 | <0.001 |
| province BC: holiday- other statutory | -0.31 | 1.12 | 0.780 |

BC as reference

| Parameter | Estimate | Standard error | p-value |
| --- | --- | --- | --- |
| intercept | 61.48 | 0.86 | <0.001 |
| post | -17.13 | 2.47 | <0.001 |
| province Ontario | 19.98 | 1.09 | <0.001 |
| time- pre-pandemic | -0.01 | 0.01 | 0.251 |
| wild | -21.29 | 2.41 | <0.001 |
| lag 1 | 1.32 | 0.41 | 0.041 |
| poly (time post, 2) 1 | 65.54 | 13.85 | <0.001 |
| poly (time post, 2) 2 | -49.14 | 8.60 | 0.003 |
| holiday- last week of December | 4.74 | 2.17 | <0.001 |
| holiday first week of January | 3.84 | 2.12 | 0.004 |
| holiday- other statutory | 0.02 | 0.79 | <0.001 |
| province Ontario: post | -16.88 | 2.55 | <0.001 |
| province Ontario: time pre | -0.04 | 0.01 | 0.310 |
| province Ontario: wild | -9.77 | 3.09 | <0.001 |
| province Ontario: poly (time post, 2) 1 | 75.00 | 16.66 | <0.001 |
| province Ontario: poly (time post, 2) 2 | -49.21 | 14.41 | 0.098 |
| province Ontario: holiday- last week of December | 3.28 | 3.06 | <0.001 |
| province Ontario: holiday- first week of January | -45.13 | 2.96 | <0.001 |
| province Ontario: holiday- other statutory | 0.31 | 1.1 | 0.062 |

Interrupted time series (ITS) plot for emergency department visits in Ontario and British Columbia (BC)


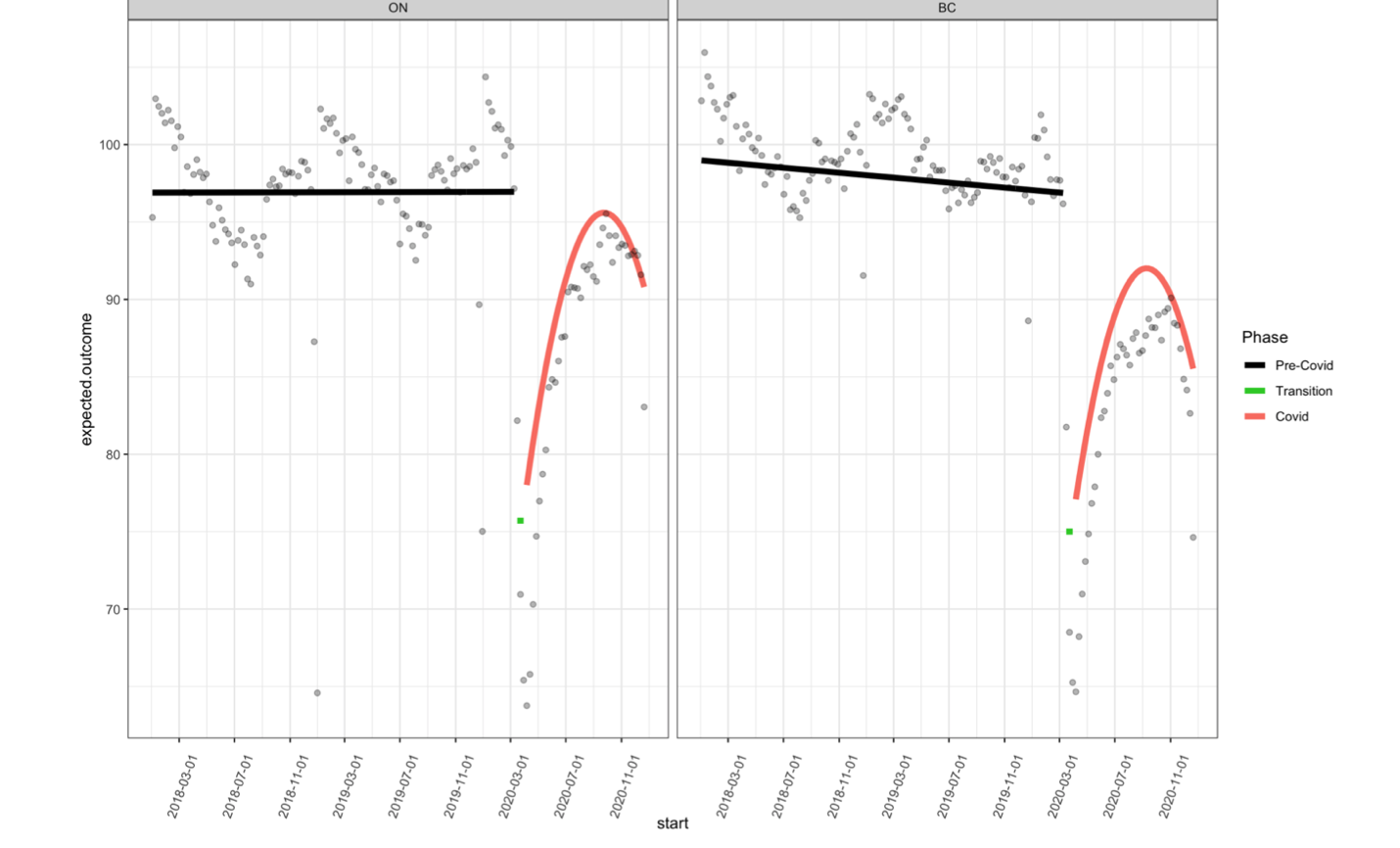


Notes: The figure shows weekly data points and linear mixed effects regression (LMER)-based expected values, conditional on the estimated ISO week level random intercepts.

The transition point represents the “wild” coefficient.

S5 Table. Interrupted time series model and plot for hospital admissions per 10,000 person-weeks in Ontario and British Columbia (BC). Please see the notes below S1 Table for parameter descriptions.

Ontario as reference

| Parameter | Estimate | Standard error | p-value |
| --- | --- | --- | --- |
| intercept | 14.69 | 0.14 | <0.001 |
| post | -4.07 | 0.35 | <0.001 |
| province BC | 3.51 | 1.09 | <0.001 |
| time- pre-pandemic | -0.05 | 0.19 | <0.001 |
| wild | -4.38 | 0.39 | <0.001 |
| lag 1 | 0.06 | 0.05 | 0.296 |
| poly (time post, 2) 1 | 21.42 | 2.22 | <0.001 |
| poly (time post, 2) 2 | -8.02 | 1.32 | <0.001 |
| holiday- last week of December | -2.30 | 0.38 | <0.001 |
| holiday- first week of January | -7.96 | 0.37 | <0.001 |
| holiday- other statutory | -0.63 | 0.14 | <0.001 |
| province BC: post | 0.35 | 0.45 | 0.434 |
| province BC: time pre | 0.00 | 0.00 | 0.788 |
| province BC: wild | -0.71 | 0.54 | 0.192 |
| province BC: poly (time post, 2) 1 | -3.33 | 2.93 | 0.256 |
| province BC: poly (time post, 2) 2 | -0.33 | 1.83 | 0.858 |
| province BC: holiday- last week of December | -0.26 | 0.54 | 0.629 |
| province BC: holiday- first week of January | 7.36 | 0.52 | <0.001 |
| province BC: holiday- other statutory | -0.15 | 0.20 | 0.431 |

BC as reference

| Parameter | Estimate | Standard error | p-value |
| --- | --- | --- | --- |
| intercept | 18.19 | 0.14 | <0.001 |
| post | -3.72 | 0.38 | <0.001 |
| province Ontario | -3.51 | 0.19 | <0.001 |
| time- pre-pandemic | -0.01 | 0.00 | 0.001 |
| wild | -5.09 | 0.41 | <0.001 |
| lag 1 | 0.06 | 0.05 | 0.296 |
| poly (time post, 2) 1 | 18.09 | 2.31 | <0.001 |
| poly (time post, 2) 2 | -8.35 | 1.37 | <0.001 |
| holiday- last week of December | -2.56 | 0.38 | <0.001 |
| holiday- first week of January | -0.63 | 0.39 | 0.104 |
| holiday- other statutory | -0.78 | 0.14 | <0.001 |
| province Ontario: post | -0.35 | 0.45 | 0.434 |
| province Ontario: time pre | -0.00 | 0.00 | 0.788 |
| province Ontario: wild | 0.71 | 0.54 | 0.192 |
| province Ontario: poly (time post, 2) 1 | 3.33 | 2.93 | 0.256 |
| province Ontario: poly (time post, 2) 2 | 0.33 | 1.83 | 0.858 |
| province Ontario: holiday- last week of December | 0.26 | 0.54 | 0.629 |
| province Ontario: holiday- first week of January | -7.33 | 0.52 | <0.001 |
| province Ontario: holiday- other statutory | 0.15 | 0.20 | 0.431 |

Interrupted time series (ITS) plot for hospital admissions in Ontario and British Columbia (BC)


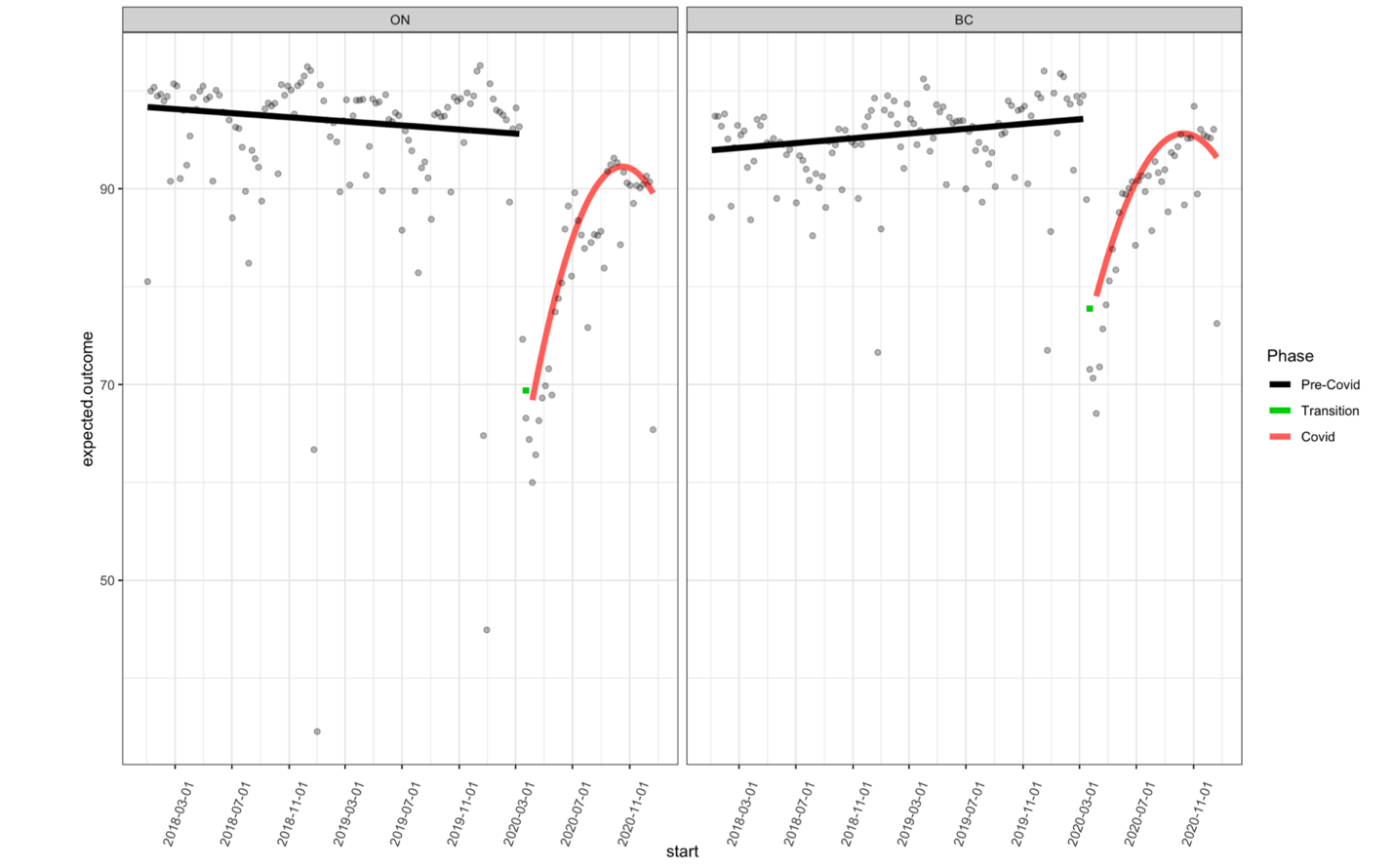


Notes: The figure shows weekly data points and linear mixed effects regression (LMER)-based expected values, conditional on the estimated ISO week level random intercepts.

The transition point represents the “wild” coefficient.

S6 Table. Interrupted time series model and plot for total healthcare costs per 10,000 person-weeks in Ontario and British Columbia (BC). Please see the notes below S1 Table for parameter descriptions.

Ontario as reference

| Parameter | Estimate | Standard error | p-value |
| --- | --- | --- | --- |
| intercept | 583325.71 | 4233.73 | <0.001 |
| post | -161407.09 | 10917.49 | <0.001 |
| province BC | -175699.92 | 5336.75 | <0.001 |
| time- pre-pandemic | -145.24 | 49.19 | 0.003 |
| wild | -154923.43 | 11327.95 | <0.001 |
| lag 1 | 5332.42 | 1946.97 | 0.007 |
| poly (time post, 2) 1 | 827978.21 | 67709.26 | <0.001 |
| poly (time post, 2) 2 | -235430.69 | 38729.88 | <0.001 |
| holiday- last week of December | -40003.95 | 4311.09 | <0.001 |
| holiday- first week of January | -243423.16 | 14061.27 | <0.001 |
| holiday- other statutory | -40003.95 | 4311.09 | <0.001 |
| province BC: post | 88717.78 | 12369.76 | <0.001 |
| province BC: time pre | 256.49 | 69.96 | <0.001 |
| province BC: wild | 76362.15 | 14801.05 | <0.001 |
| province BC: poly (time post, 2) 1 | -438051.61 | 80878.09 | <0.001 |
| province BC: poly (time post, 2) 2 | 107437.55 | 50166.30 | 0.033 |
| province BC: holiday- last week of December | 88063.86 | 14742.35 | <0.001 |
| province BC: holiday- first week of January | 222078.77 | 14316.26 | <0.001 |
| province BC: holiday- other statutory | 21475.33 | 5397.42 | <0.001 |

BC as reference

| Parameter | Estimate | Standard error | p-value |
| --- | --- | --- | --- |
| intercept | 407625.79 | 4697.26 | <0.001 |
| post | -72689.31 | 12022.50 | <0.001 |
| province Ontario | 175699.92 | 5336.75 | <0.001 |
| time- pre-pandemic | 111.25 | 50.02 | 0.027 |
| wild | -78561.29 | 11450.92 | <0.001 |
| lag 1 | 5332.42 | 1946.97 | 0.007 |
| poly (time post, 2) 1 | 389926.60 | 73264.75 | <0.001 |
| poly (time post, 2) 2 | 127993.14 | 39748.73 | 0.002 |
| holiday- last week of December | -89431.09 | 13378.32 | <0.001 |
| holiday first week of January | -21344.39 | 14499.38 | 0.144 |
| holiday- other statutory holiday | -18528.62 | 4309.02 | <0.001 |
| province Ontario: post | -88717.78 | 12369.76 | <0.001 |
| province Ontario: time pre | -256.49 | 69.96 | 0.310 |
| province Ontario: wild | -76362.15 | 14801.05 | <0.001 |
| province Ontario: poly (time post, 2) 1 | 438051.61 | 80878.09 | <0.001 |
| province Ontario: poly (time post, 2) 2 | -107437.55 | 50166.30 | 0.033 |
| province Ontario: holiday- last week of December | -88063.86 | 14742.35 | <0.001 |
| province Ontario: holiday- first week of January | -222078.77 | 14316.26 | <0.001 |
| province Ontario: holiday- other statutory | -21475.33 | 5397.42 | <0.001 |

Interrupted time series (ITS) plot for total healthcare costs in Ontario and British Columbia (BC)


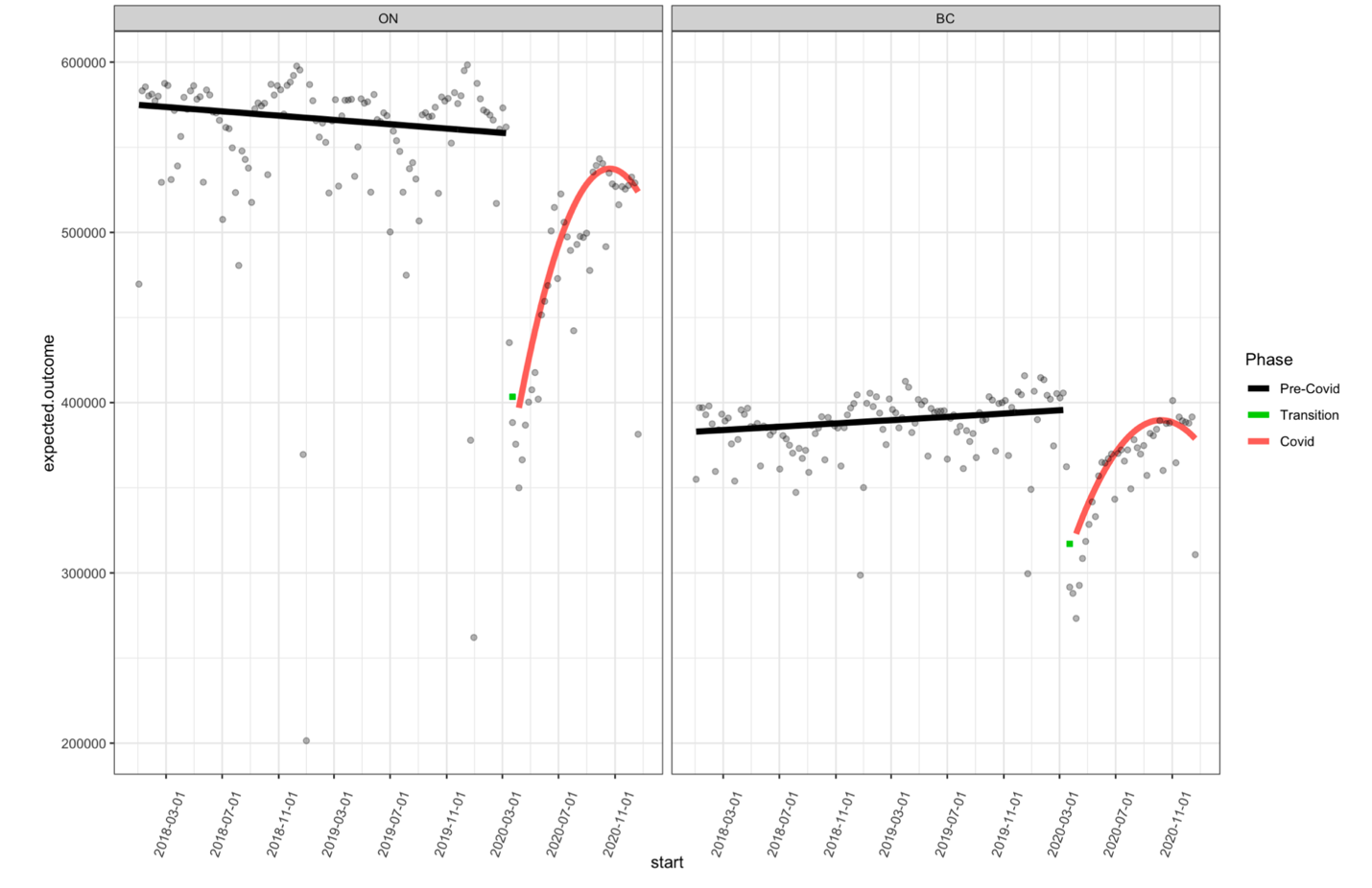


Notes: The figure shows weekly data points and linear mixed effects regression (LMER)-based expected values, conditional on the estimated ISO week level random intercepts.

The transition point represents the “wild” coefficient.

S7 Table. Total and resource-specific absolute costs (2020 Canadian dollars) per quarter for all individuals in Ontario, 2018 and 2019. For readability, all costs are rounded to the nearest thousand dollars.

|  | **Time period^1^** | | | | | | | |
| --- | --- | --- | --- | --- | --- | --- | --- | --- |
| **Health Resource** | **2018 Q1** | **2018 Q2** | **2018 Q3** | **2018 Q4** | **2019 Q1** | **2019 Q2** | **2019 Q3** | **2019 Q4** |
| Hospitalization | 2,383,710,000 | 2,298,598,000 | 2,228,487,000 | 2,335,211,000 | 2,344,001,000 | 2,360,386,000 | 2,277,098,000 | 2,357,470,000 |
| Emergency Department visits | 457,292,000 | 434,485,000 | 437,702,000 | 434,953,000 | 417,637,000 | 462,826,000 | 459,158,000 | 458,680,000 |
| Same Day Surgery | 386,010,000 | 412,998,000 | 362,190,000 | 398,285,000 | 388,633,000 | 437,001,000 | 387,282,000 | 420,493,000 |
| Physician services- PCPs | 612,903,000 | 607,696,000 | 585,927,000 | 630,475,000 | 577,781,000 | 619,588,000 | 590,315,000 | 626,690,000 |
| Physician services - specialists | 1,776,105,000 | 1,873,709,000 | 1,752,995,000 | 1,837,692,000 | 1,706,931,000 | 1,884,179,000 | 1,779,826,000 | 1,846,542,000 |
| Non-physician services^2^ | 41,386,000 | 46,023,000 | 44,935,000 | 46,376,000 | 39,746,000 | 46,552,000 | 45,296,000 | 46,152,000 |
| Cancer clinics | 287,396,000 | 337,766,000 | 331,431,000 | 337,375,000 | 315,902,000 | 389,003,000 | 380,853,000 | 385,587,000 |
| Dialysis clinics | 185,820,000 | 189,827,000 | 190,008,000 | 192,799,000 | 174,142,000 | 193,612,000 | 194,617,000 | 197,042,000 |
| Outpatient clinics^3^ | 751,801,000 | 809,801,000 | 746,343,000 | 791,808,000 | 737,167,000 | 818,212,000 | 754,725,000 | 793,864,000 |
| Laboratory tests^4^ | 254,789,000 | 270,952,000 | 250,115,000 | 252,488,000 | 162,225,000 | 181,059,000 | 170,715,000 | 170,282,000 |
| Outpatient drugs^5^ | 1,604,589,000 | 1,658,137,000 | 1,545,305,000 | 1,688,781,000 | 1,562,548,000 | 1,643,990,000 | 1,546,498,000 | 1,746,544,000 |
| NDFP drugs^6^ | 105,388,000 | 115,152,000 | 117,430,000 | 120,198,000 | 121,979,000 | 135,941,000 | 147,624,000 | 147,712,000 |
| Home Care | 879,826,000 | 899,728,000 | 853,465,000 | 891,455,000 | 890,451,000 | 916,882,000 | 893,434,000 | 914,144,000 |
| Long-term Care | 666,947,000 | 672,590,000 | 655,315,000 | 630,350,000 | 584,725,000 | 576,725,000 | 546,319,000 | 506,937,000 |
| Complex Continuing Care | 209,414,000 | 208,709,000 | 209,354,000 | 212,270,000 | 210,018,000 | 212,899,000 | 209,315,000 | 207,729,000 |
| Mental health inpatient | 169,219,000 | 174,008,000 | 174,184,000 | 172,640,000 | 169,027,000 | 167,297,000 | 166,155,000 | 165,326,000 |
| TOTAL | 10,772,595,000 | 11,010,180,000 | 10,485,184,000 | 10,973,157,000 | 10,402,913,000 | 11,046,153,000 | 10,549,231,000 | 10,991,194,000 |

^1^ Time periods comprise 13 weeks in each ISO year

^2^ Non-physician services covered by the Ontario Health Insurance Plan include care provided by optometrists, podiatrists, physiotherapists, and other allied health professionals, for eligible individuals [18]

^3^ Outpatient hospital clinics, other than dialysis and cancer

^4^ Excludes SARS-CoV-2 tests

^5^ Outpatient prescription drugs for eligible individuals, aged 65 years and older or who meet other criteria [19]

^6^ The New Drug Funding Program reimburses cancer centres for expensive intravenous cancer chemotherapy [16, 20].

Abbreviations: NDFP- New Drug Funding Program; PCP- primary care provider; Q- quarter

S8 Table. Total and resource-specific absolute costs (2020 Canadian dollars) per quarter for all individuals in British Columbia (BC), 2018 and 2019. For readability, all costs are rounded to the nearest thousand dollars.

|  | **Time period^1^** | | | | | | | |
| --- | --- | --- | --- | --- | --- | --- | --- | --- |
| **Health Resource** | **2018 Q1** | **2018 Q2** | **2018 Q3** | **2018 Q4** | **2019 Q1** | **2019 Q2** | **2019Q3** | **2019 Q4** |
| Hospitalization | 994,358,000 | 974,734,000 | 944,670,000 | 974,220,000 | 1,042,679,000 | 1,032,987,000 | 1,006,627,000 | 1,022,109,4000 |
| Emergency Department visits | 121,489,000 | 120,645,000 | 119,614,000 | 118,582,000 | 122,145,000 | 122,391,000 | 121,374,000 | 121,251,000 |
| Same Day Surgery | 164,689,000 | 171,334,000 | 156,695,000 | 166,361,000 | 177,226,000 | 181,977,000 | 167,745,000 | 176,406,000 |
| Physician services – PCPs | 272,730,000 | 271,313,000 | 255,566,000 | 263,566,000 | 275,979,000 | 282,039,000 | 268,984,000 | 273,531,000 |
| Physician services - specialists | 189,238,000 | 205,074,000 | 189,080,000 | 200,580,000 | 202,975,000 | 217,258,000 | 202,037,000 | 211,126,000 |
| Other MSP^2^ | 438,399,000 | 437,826,000 | 405,967,000 | 416,819,000 | 459,475,000 | 455,710,000 | 425,752,000 | 434,489,000 |
| Outpatient prescription drugs | 741,947,000 | 783,729,000 | 781,654,000 | 806,508,000 | 781,455,000 | 814,618,000 | 802,898,000 | 838,844,000 |
| Home Care | 148,803,000 | 157,320,000 | 146,866,000 | 147,384,000 | 135,942,000 | 160,280,000 | 151,670,000 | 153,799,000 |
| Total costs | 3,071,653,000 | 3,121,975,000 | 3,000,112,000 | 3,094,019,000 | 3,197,876,000 | 3,267,260,000 | 3,147,087,000 | 3,231,556,000 |

^1^ Time periods comprise 13 weeks in each ISO year

^2^ Other MSP include Medical Services Plan records for diagnostic procedures, procedures and other services

Abbreviations: MSP- Medical Services Plan; PCP- primary care provider; Q- quarter

S9 Table. Total absolute costs (in 2020 Canadian dollars) in 2020 for the full population and for individuals who tested positive for SARS-Cov-2 in Ontario and British Columbia (BC). For readability, costs are rounded to the nearest thousand dollars; the percentages were calculated using costs estimated to the dollar.

|  | Ontario | | British Columbia | |
| --- | --- | --- | --- | --- |
| Group | Total 2020 cost | % of cost for full population | Total 2020 cost | % of cost for full population |
| Acute-COVID-19 | 265,394,000 | 0.69 | 60,165,000 | 0.49 |
| Post-acute-COVID-19 | 238,550,000 | 0.62 | 29,598,000 | 0.24 |
| All COVID-19 | 503,944,000 | 1.31 | 89,762,000 | 0.72 |
| Non-COVID-19 | 37,893,153,000 | 98.69 | 12,304,119,000 | 99.3 |
| Full population | 38,397,097,000 | 100.0 | 12,393,881,000 | 100.0 |

Acute COVID-19 was defined as the first 4 weeks (inclusive) after the positive test.

Post-acute COVID-19 was defined from the end of the acute COVID-19 period to end of observation.

S1 Figure. Number of cancer clinic visits in Ontario per 10,000 person-weeks in each ISO week from January 1, 2018 to December 27, 2020

The vertical line indicates the week of March 16 to 22, 2020.

S2 Figure. Number of a) home care services in Ontario and British Columbia, and b) dialysis clinic visits in Ontario per 10,000 person-weeks in each ISO week from January 1, 2018 to December 27, 2020.

The vertical line indicates the week of March 16 to 22, 2020.

1. Home care services

1. Dialyisis clinic visits (Ontario)

**References**

1. Government of Ontario. Databases. Registered Persons Database (RPDB). Available from: <https://data.ontario.ca/dataset/registered-persons-database-rpdb>.

2. British Columbia Ministry of Health [creator]. Consolidation file (MSP Registration and Premium Billing). V2. Population Data BC [publisher]. Data Extract. MOH (2020); from <http://www.popdata.bc.ca/data>.

3. Ontario Health, Appendix 1: Case Definitions and Disease-Specific Information- Disease: Diseases caused by a novel coronavirus, including Coronavirus Disease 2019 (COVID-19), Severe Acute Respiratory Syndrome (SARS) and Middle East Respiratory Syndrome (MERS), Available from: <https://www.health.gov.on.ca/en/pro/programs/publichealth/coronavirus/docs/2019_case_definition.pdf>, Effective June 2022.

4. British Columbia Ministry of Health [creator]. BC COVID-19 Test Lab Data. Population Data BC [publisher]. Data Extract. MOH (2020), from <http://www.popdata.bc.ca/data>.

5. Canadian Institute for Health Information. Discharge Abstract Database metadata (DAD). Available from: <https://www.cihi.ca/en/discharge-abstract-database-metadata-dad>.

6. Canadian Institute for Health Information [creator] (2020). Discharge Abstract Database (Hospital Separations). V2. Population Data BC [Publisher]. Data Extract. MOH (2020). from <http://www.popdata.bc.ca/data>.

7. Canadian Institute for Health Information. National Ambulatory Care Reporting System metadata (NACRS). Available from: <https://www.cihi.ca/en/national-ambulatory-care-reporting-system-metadata-nacrs>.

8. Canadian Institute for Health Information [creator] (2020). National Ambulatory Care Reporting System. V2. Population Data BC [Publisher}. Data Extract. MOH (2020). from <http://www.popdata.bc.ca/data>..

9. Government of Ontario. Databases. Ontario Drug Benefit (ODB) Database. Available from: <https://data.ontario.ca/dataset/ontario-drug-benefit-odb-database>.

10. British Columbia Ministry of Health [creator] (2020). PharmaNet. V2. BC Ministry of Health [Publisher]. Data Extract. Data Stewardship Committee (2020). from <http://www.popdata.bc.ca/data>.

11. Government of Ontario. Data catalogue. Claims History Database. Available from: <https://data.ontario.ca/en/dataset/claims-history-database>.

12. British Columbia Ministry of Health [creator.] (2020). Medical Services Plan (MSP) Payment Information File. V2. Population Data BC [Publisher]. Data Extract. MOH (2020). from <http://www.popdata.bc.ca/data>.

13. Government of Ontario. Databases. Home Care Database (HCD). Available from: <https://data.ontario.ca/en/dataset/home-care-data-hcd>.

14. Canadian Institute for Health Information. Continuing Care Metadata. Available from: https://www.cihi.ca/en/continuing-care-metadata#:~:text=The%20Continuing%20Care%20Reporting%20System,term%20care%20homes%20in%20Canada.

15. British Columbia Ministry of Health [creator] (2020). Home & Community Care (Continuing Care). V2. Population Data BC [Publisher]. Data Extract. MOH (2020). from <http://www.popdata.bc.ca/data>.

16. Cancer Care Ontario. The New Drug Funding Program, Available fromt: <https://www.cancercareontario.ca/en/Funding/New_Drug_Funding_Program>.

17. Benchimol EI, Smeeth L, Guttmann A, Harron K, Moher D, Petersen I,et al. The REporting of studies Conducted using Observational Routinely-collected health Data (RECORD) Statement. PLoS Med. 2015;12: e1001885.

18. Ontario Health. What OHIP covers. Published April 21, 2017; updated October 21, 2021.

Available from: <https://www.ontario.ca/page/what-ohip-covers>.

19. Ontario Health. Get coverage for prescription drugs. Published September 20,2016; updated May 31, 2022. Available from:

<https://www.ontario.ca/page/get-coverage-prescription-drugs>,

20. Evans WK, Nefsky M, Pater J, Browman G, Cowan DH. Cancer Care Ontario's New Drug Funding Program: controlled introduction of expensive anticancer drugs. Chron Dis Can. 2002;23: 152-156.
